# Burden of metabolic diseases in mainland China from 1990 to 2021: results from the global burden of disease study 2021

**DOI:** 10.3389/fpubh.2026.1846594

**Published:** 2026-07-14

**Authors:** Mutian Niu, Yiming Sun, Yan Chen, Ran Qi, Yangxi Hu, Ping Wang, Yingjian Zhang

**Affiliations:** 1 Department of Gastroenterology, The First Affiliated Hospital of Henan University of Science and Technology, Luoyang, China; 2 Department of Gastrointestinal Surgery, Zhengzhou Central Hospital Affiliated to Zhengzhou University, Zhengzhou, China; 3 School of Basic Medicine and Forensic Medicine, Henan University of Science and Technology, Luoyang, China

**Keywords:** global burden of disease, hypertension, metabolic diseases, metabolic dysfunction-associated steatotic liver disease, obesity

## Abstract

**Background:**

Within the last three decades, common metabolic diseases have become a worldwide health burden, including type 2 diabetes mellitus (T2DM), hypertension, obesity, hypercholesterolemia, and metabolic dysfunction-associated steatotic liver disease (MASLD). However, trends and burdens of these metabolic diseases across age and sex in Mainland China from 1990 to 2021 remain unclear.

**Methods:**

Estimated disability-adjusted life years (DALYs) and deaths from GBD 2021 were utilized to analyze common metabolic diseases (T2DM, hypertension, obesity, hypercholesterolemia, and MASLD). Trend analyses were conducted using age-standardized DALYs (mortality) per 100,000 population and the total percentage change (TPC) between 1990 and 2021. Estimates were presented with uncertainty intervals (UI).

**Results:**

In 2021, among the five common metabolic diseases, hypertension had the highest burden (55.69 million [95% UI: 43.30–70.33] DALYs), followed by obesity (20.87 million [95% UI: 8.50–33.64] DALYs), hypercholesterolemia (18.41 million [95% UI: 10.27–27.11]), T2DM (11.47 million [95% UI: 8.83–14.70] DALYs) and MASLD (0.41 million [95% UI: 0.32–0.53] DALYs). Over the past three decades, the burden of these metabolic diseases has increased, albeit at varying magnitudes (ranging from a 0.64-fold to a 2.84-fold increase). Obesity and T2DM were the top two diseases with the highest TPC (2.84 and 1.90 respectively). The burden of DALYs and deaths, as well as their TPC in males with almost all the five common metabolic diseases was higher than that in females. In addition, the 55–74 years age group had the greatest burden of metabolic conditions.

**Conclusion:**

The burden of common metabolic diseases remained high and continued to increase in Mainland China over the past 30 years. Therefore, urgent interventions are needed to address the concerning surge in DALYs and mortality as well as the persistent age-sex disparities attributed to metabolic diseases.

## 1 Introduction

Metabolic diseases refer to a spectrum of common metabolic dysregulation including hypertension, type 2 diabetes mellitus (T2DM), hyperlipidemia, obesity, and metabolic dysfunction-associated steatotic liver disease (MASLD) (1, 2). These diseases often occur simultaneously or successively, and are associated with increased risks of death from cardiovascular and cerebrovascular diseases (3, 4). Recently, the Global Burden of Disease (GBD) 2021 risk factor analysis observed a 49.4% increase in disability-adjusted life-years (DALYs) attributable to metabolic risks, while the other two level 1 risk factors (environmental and occupational risks, behavioral risks) declined from 2000 to 2021 (5). Furthermore, another recent study reported that the global burden of the five metabolic diseases has continued to increase over the past three decades, and Mainland China ranks among the top two regions with the highest absolute burden of each disease (1). The Global Burden of Disease, Injuries, and Risk Factors Study (GBD) 2021 data, updated on May 16, 2024, provides global age-sex-region-specific estimates of the risk factors and causes of death, which thus can be used to characterize the temporal burden of metabolic diseases in China (5). Since hypertension, obesity, and hypercholesterolemia are classified as risk factors rather than diseases in the GBD data, previous GBD studies have focused only on some kinds of disease burden attributable to metabolic risk factors in China (6, 7). However, the total burden caused by metabolic risk factors as well as their comparison in China remains poorly understood. Based on the latest GBD data for 2021, our study aims to provide insights into the trends and burdens of common metabolic diseases and risk factors in China from 1990 to 2021 for the first time, highlighting differences by age and sex.

## 2 Methods

### 2.1 Data source

Estimates from the GBD 2021 study were used for the analysis of trends in incidence, death, DALYs and total percentage change (TPC) of metabolic diseases and risk factors from 1990 to 2021 (5, 8). Nation-level data on incidence, mortality, DALYs and TPC indicators for common metabolic diseases between 1990 and 2021 were extracted using the Global Health Data Exchange (GHDx) query tool.^1^ In the GBD 2021, estimates of incidence, prevalence, DALYs, and mortality are provided for T2DM and the overall disease burden attributable to MASLD. In contrast, only DALYs and mortality are available for hypertension, obesity, and hypercholesterolemia, as these are categorized as risk factors rather than distinct diseases in the GBD framework. To harmonize the indicators in the text, DALYs and mortality for each metabolic disease and risk factor were analyzed, and these three metabolic risk factors were collectively referred to as metabolic diseases in this study for narrative consistency. As per the GBD algorithm, each rate is reported per 100,000 population, and estimates in this study were presented with 95% uncertainty intervals (UI).

### 2.2 Definition

The common metabolic diseases studied in our study include T2DM, hypertension, obesity, hypercholesterolemia and MASLD, which are defined by GBD 2021 official terms as T2DM, high systolic blood pressure, high body-mass index, high LDL cholesterol, and MASLD, respectively. The definitions and diagnostic criteria for metabolic diseases and risk factors were described in the previous study (2). As a specific indicator for comparing mortality rates and nonfatal outcomes within and between diseases, DALYs are calculated as the sum of years lived with disability (YLDs) and years of life lost (YLLs) (9). The percentage of relative changes in deaths and DALYs between 1990 and 2021 was used to examine changing trends of metabolic diseases and risk factors, which was calculated in the GBD database by subtracting 1990 estimates from 2021 estimates and dividing the difference by 1990 estimates. If both 95% UIs were >0, the trend was identified as increasing, and if both 95% UIs were <0, the trend was identified as decreasing. All percentage change (total percentage change, TPC) values for deaths and DALYs between 1990 and 2021 in this study were presented in decimal form instead of percentage format. All analyses were conducted using R 4.4.1 and GraphPad Prism 9.

## 3 Results

### 3.1 Summary of results in 2021

**Figure 1:**
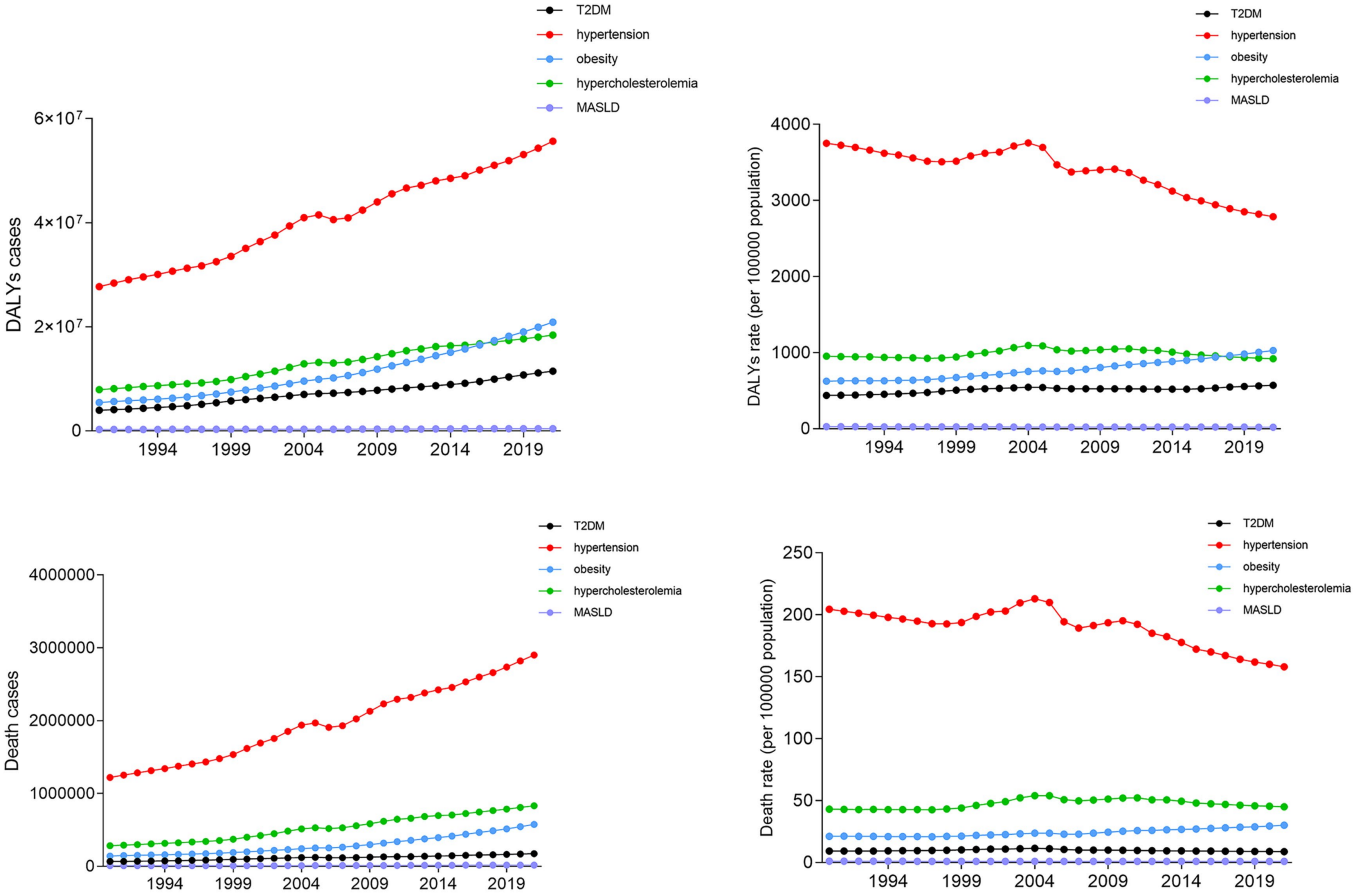
Burden of metabolic diseases by age group in Mainland China, 2021. **(A)** DALYs cases; **(B)** DALYs rate; **(C)** Death cases; **(D)** Death rate.

**Figure 2:**
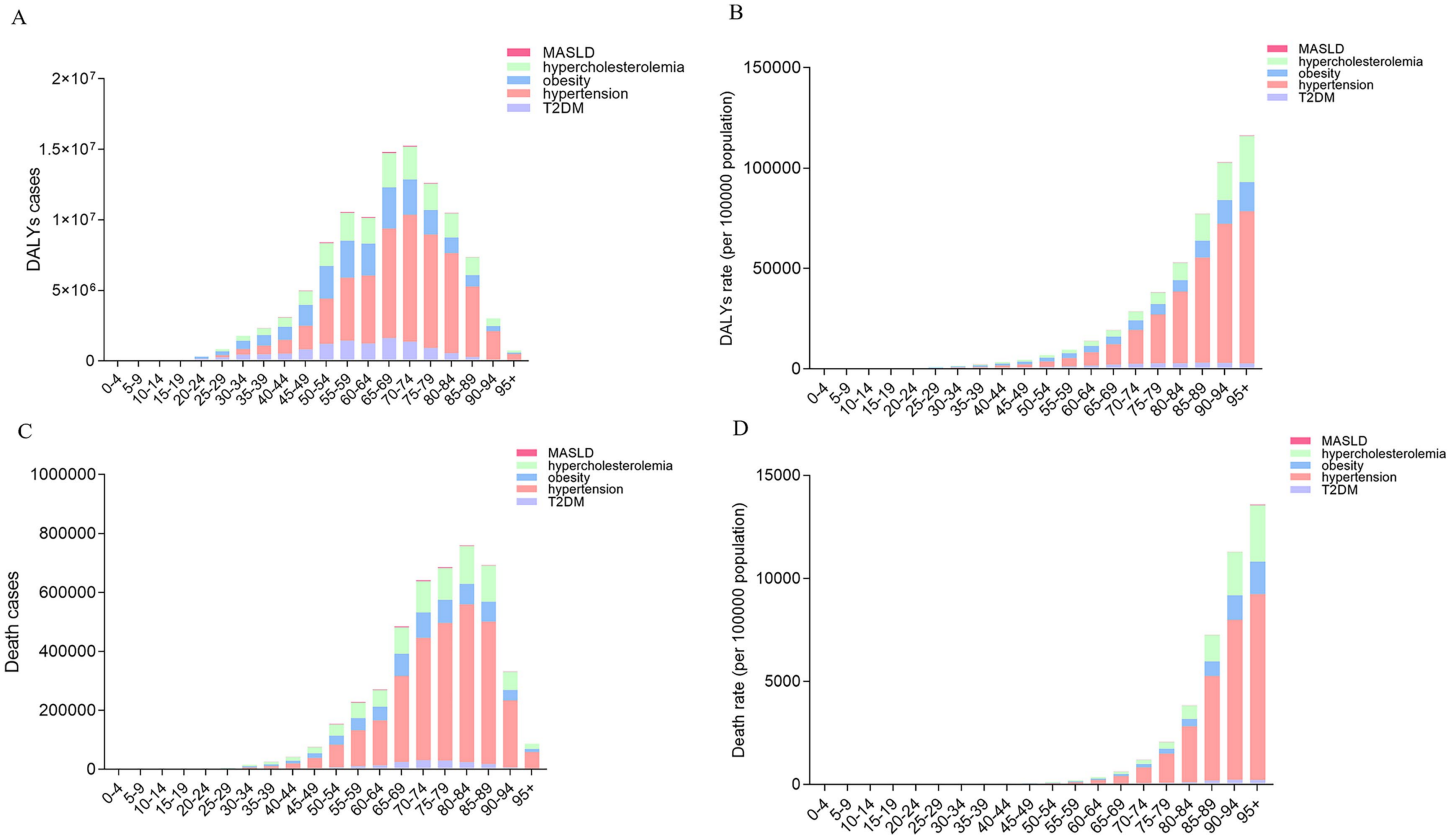
Temporal trends of metabolic diseases in Mainland China, 1990–2021. **(A)** DALYs cases; **(B)** age-standardized DALYs rate; **(C)** Death cases; **(D)** age-standardized death rate.

In 2021, the total number of incident cases of T2DM was 3.97 million (95% UI: 3.57–4.41) in Mainland China, resulting in 11.47 million (95% UI: 8.83–14.70) DALYs. The total number of incident cases of MASLD was 9.57 million (95% UI: 8.79–10.38), resulting in 0.41 million (95% UI: 0.32–0.53) DALYs. Due to their classification as risk factors rather than diseases in GBD, prevalence estimates for hypertension, obesity and hypercholesterolemia were unavailable. For hypertension, DALYs were estimated at 55.69 million (95% UI: 43.30–70.33), for obesity at 20.87 million (95% UI: 8.50–33.64), and for hypercholesterolemia at 18.41 million (95% UI: 10.27–27.11). Therefore, the highest DALYs number for metabolic disorders was observed in hypertension, followed by obesity, hypercholesterolemia, T2DM, and MASLD (Figures 1A, 2A; Tables 1–3). The age-standardized DALYs rates of these five conditions in 2021 were ranked in the same order as the number of DALYs (Figures 1B, 2B; Tables 1–3).

**Table 1:** Trends of metabolic diseases in number of DALYs and age-standardized DALYs in China, from 1990 to 2021: overall population.

Diseases	Number of DALYs (95% UI)			Age-standardized DALYs rate (95% UI)		
	1990	2021	TPC (1990–2021)	1990	2021	TPC (1990–2021)
T2DM	3,960,056.97 (3,188,322.11–4,872,251.94)	11,465,017.87 (8,834,150.96–14,700,712.03)	1.9 (1.62–2.19)	438.68 (358.49–531.54)	569.84 (435.43–734.18)	0.3 (0.17 to 0.43)
Hypertension	27,723,996.31 (21,005,923.13–34,610,131.92)	55,685,409.75 (43,295,923.69–70,330,947.63)	1.01 (0.61–1.52)	3,753.46 (2,897.55–4,650.92)	2,788.47 (2,170.34–3,503.6)	−0.26 (−0.4 to 0.07)
Obesity	5,433,920.19 (3,170,338.21–8,612,166.96)	20,865,171.63 (8,495,257.65–33,636,820.3)	2.84 (1.67–3.58)	624.53 (366.98–975.46)	1,028.85 (428.39–1,635.04)	0.65 (0.12 to 1)
Hypercholesterolemia	7,905,011.57 (4,442,434.31–11,142,363.83)	18,407,758.53 (10,268,060.98–27,106,770.94)	1.33 (0.87–1.88)	953.85 (511.6–1,397.48)	920.69 (511.71–1,359.77)	−0.03 (−0.21 to 0.2)
MASLD	253,395.72 (193,645.55–317,928.54)	414,471.24 (317,240.43–530,168.13)	0.64 (0.28–1.14)	27.38 (21.16–34.32)	19.83 (15.29–24.98)	−0.28 (−0.43 to 0.05)

DALYs, disability-adjusted life years; TPC, total percentage change; UI, uncertainty interval; T2DM, type 2 diabetes mellitus; MASLD, metabolic dysfunction-associated steatotic liver disease.

**Table 2:** Trends of metabolic diseases in number of DALYs and age-standardized DALYs in China, from 1990 to 2021: males.

Diseases and sex	Number of DALYs (95% UI)			Age-standardized DALYs rate (95% UI)		
	1990	2021	TPC (1990–2021)	1990	2021	TPC (1990–2021)
T2DM						
Male	1,923,430.17 (1,506,786.27–2,376,228.52)	6,043,946.99 (4,635,122.64–7,840,311.45)	2.14 (1.75–2.55)	429.18 (343.3–523.73)	620.06 (473.83–801.09)	0.44 (0.27 to 0.62)
Hypertension						
Male	14,701,126.54 (10,638,928.45–18,979,771.42)	32,585,821.91 (23,720,529.49–43,619,331.56)	1.22 (0.6–2.06)	4,187.02 (3,065.92–5,259.14)	3,582.86 (2,653.82–4,720.82)	−0.14 (−0.37 to 0.15)
Obesity						
Male	2,569,459.42 (1,454,890.32–3,936,336.04)	10,376,055.26 (4,758,114.76–17,052,144.21)	3.04 (1.9–4.07)	598.18 (354–901.25)	1,079.27 (508.29–1,769.82)	0.8 (0.21 to 1.3)
Hypercholesterolemia						
Male	4,375,007.6 (2,402,731.02–6,203,410.37)	11,170,922.17 (6,060,075.25–16,715,660.77)	1.55 (0.84–2.38)	1,072.92 (561–1,571.67)	1,193.16 (635.37 to 1,787.06)	0.11 (−0.17–0.43)
MASLD						
Male	135,110.26 (101,287.25–176,547.87)	231,772.24 (163,850.63–317,664.85)	0.72 (0.23–1.36)	28.41 (21.37–37.25)	22.93 (16.59–31.03)	−0.19 (−0.42 to 0.11)

DALYs, disability-adjusted life years; TPC, total percentage change; UI, uncertainty interval; T2DM, type 2 diabetes mellitus; MASLD, metabolic dysfunction-associated steatotic liver disease.

**Table 3:** Trends of metabolic diseases in number of DALYs and age-standardized DALYs in China, from 1990 to 2021: females.

Diseases and sex	Number of DALYs (95% UI)			Age-standardized DALYs rate (95% UI)		
	1990	2021	TPC (1990–2021)	1990	2021	TPC (1990–2021)
T2DM						
Female	2,036,626.81 (1,636,450.48–2,518,869.61)	5,421,070.88 (4,185,594.85–6,949,687.55)	1.66 (1.35–2.01)	450.1 (365.37–553.16)	523.52 (401.27–676.86)	0.16 (0.01 to 0.32)
Hypertension						
Female	13,022,869.76 (9,581,262.8–16,714,782.35)	23,099,587.83 (17,245,046.17–30,777,508.68)	0.77 (0.31–1.49)	3,391.66 (2,537.97–4,296.76)	2,142.69 (1,581.73–2,850.76)	−0.37 (−0.52 to 0.14)
Obesity						
Female	2,864,460.77 (1,632,580.8–4,675,032.38)	10,489,116.36 (4,057,798.22–17,009,321.29)	2.66 (1.41–3.51)	653.68 (374.1–1,052.11)	984.13 (386.69–1,579.28)	0.51 (−0.02 to 0.86)
Hypercholesterolemia						
Female	3,530,003.97 (1,950,253.56–5,252,967.15)	7,236,836.36 (3,907,031.17–11,207,330.64)	1.05 (0.51–1.7)	846.75 (450.67–1,277.29)	679.55 (369.17–1,050.9)	−0.2 (−0.39 to 0.05)
MASLD						
Female	118,285.47 (86,842.13–152,248.17)	182,699 (135,754.3–245,720.8)	0.54 (0.12–1.26)	26.02 (19.19–33.51)	16.81 (12.56–22.53)	−0.35 (−0.54 to 0.05)

DALYs, disability-adjusted life years; TPC, total percentage change; UI, uncertainty interval; T2DM, type 2 diabetes mellitus; MASLD, metabolic dysfunction-associated steatotic liver disease.

For the total number of deaths in 2021, the leading cause was hypertension with 2.9 million (95% UI: 2.27–3.65), followed by hypercholesterolemia with 0.83 million (95% UI: 0.42–1.29), obesity with 0.58 million (95% UI: 0.29–0.92), T2DM with 0.17 million (95% UI: 0.14–0.21), and MASLD with 16, 748.37 (95% UI: 12, 854.61–21, 107.85; Figures 1C, 2C; Tables 4–6). As with deaths number in 2021, the burden of age-standardized deaths rate was ranked from highest to lowest as follows: hypertension, hypercholesterolemia, obesity, T2DM and MASLD (Figures 1D, 2D; Tables 4–6).

**Table 4:** Trends of metabolic diseases in number of deaths and age-standardized deaths rate in China, from 1990 to 2021: overall population.

Diseases	Number of deaths (95% UI)			Age-standardized deaths rate (95% UI)		
	1990	2021	TPC (1990–2021)	1990	2021	TPC (1990–2021)
T2DM	66,458.63 (57,966.23–75,920.69)	174,515.38 (144,849.6–207,116.82)	1.63 (1.09–2.32)	9.29 (8.16–10.49)	8.74 (7.26–10.35)	−0.06 (−0.24 to 0.17)
Hypertension	1,219,088.76 (941,839.91–1,498,885.64)	2,901,716.69 (2,267,615.37–3,650,658.98)	1.38 (0.89–2)	204.49 (158.12–249.53)	158.01 (122.81–198.51)	−0.23 (−0.37 to 0.03)
Obesity	142,048.38 (927,73.41–216,077.1)	575,624.46 (291,290.8–923,747.75)	3.05 (1.59–4.29)	21.01 (13.05–32.02)	30.01 (15.41–47.75)	0.43 (−0.11 to 0.91)
Hypercholesterolemia	282,935.07 (148,118.13–420,823.27)	832,884.39 (424,042.39–1,289,266)	1.94 (1.34–2.64)	43.1 (21.29–67.6)	44.97 (23.22–69.89)	0.04 (−0.14 to 0.28)
MASLD	8,351.31 (6,428.24–10,455.13)	16,748.37 (12,854.61–21,107.85)	1.01 (0.57–1.6)	1.04 (0.8–1.31)	0.82 (0.64–1.03)	−0.21 (−0.38 to 0.02)

TPC, total percentage change; UI, uncertainty interval; T2DM, type 2 diabetes mellitus; MASLD, metabolic dysfunction-associated steatotic liver disease.

**Table 5:** Trends of metabolic diseases in number of deaths and age-standardized deaths rate in China, from 1990 to 2021: overall population: males.

Diseases and sex	Number of deaths (95% UI)			Age-standardized deaths rate (95% UI)		
	1990	2021	TPC (1990–2021)	1990	2021	TPC (1990–2021)
T2DM						
Male	28,056.23 (23,074.6–33,629.56)	86,026.3 (66,966.42–108,719.31)	2.07 (1.22–3.18)	9.04 (7.63–10.53)	9.97 (7.83–12.45)	0.1 (−0.18 to 0.45)
Hypertension						
Male	608,732.77 (444,759.74–775,578.97)	1,614,181.01 (1,188,832.46–2,134,178.09)	1.65 (0.92–2.65)	228.8 (166.52–287.08)	207.32 (154.26–267.99)	−0.09 (−0.34 to 0.24)
Obesity						
Male	64,304.24 (42,175.19–96,760.15)	281,649.58 (143,642.63–474,054.63)	3.38 (1.82–5.09)	21.11 (12.95–31.86)	33.6 (17.67–55.75)	0.59 (0 to 1.22)
Hypercholesterolemia						
Male	148,250.19 (76,801.89–219,067.49)	472,161.65 (232,649.94–732,842.74)	2.18 (1.29–3.24)	48.82 (23.61–75.49)	58.57 (28.56–90.82)	0.2 (−0.09 to 0.54)
MASLD						
Male	4,164.85 (3,111.86–5,436.82)	8,743.63 (6,203.62–11,893.48)	1.1 (0.49–1.91)	1.04 (0.79–1.32)	0.92 (0.67–1.24)	−0.11 (−0.35 to 0.22)

TPC, total percentage change; UI, uncertainty interval; T2DM, type 2 diabetes mellitus; MASLD, metabolic dysfunction-associated steatotic liver disease.

**Table 6:** Trends of metabolic diseases in number of deaths and age-standardized deaths rate in China, from 1990 to 2021: overall population: females.

Diseases and sex	Number of deaths (95% UI)			Age-standardized deaths rate (95% UI)		
	1990	2021	TPC (1990–2021)	1990	2021	TPC (1990–2021)
T2DM						
Female	38,402.41 (31,868.87–46,135.32)	88,489.08 (70,122.5–109,406.66)	1.3 (0.74–2.06)	9.84 (8.19–11.76)	8.08 (6.42–9.95)	−0.18 (−0.37 to 0.08)
Hypertension						
Female	610,355.98 (452,537.31–772,183.37)	1,287,535.68 (961,362.69–1,738,090.02)	1.11 (0.55–1.93)	187.58 (139.83–236)	124.35 (93.01–167.13)	−0.34 (−0.51 to 0.1)
Obesity						
Female	77,744.14 (50,224.16–120,669.23)	293,974.88 (137,764.4–490,834.5)	2.78 (1.32–4.26)	21.32 (13.39–32.89)	27.63 (13.2–46.67)	0.3 (−0.24 to 0.81)
Hypercholesterolemia						
Female	134,684.88 (70,198.47–208,307.91)	360,722.74 (186,198.69–584,410.71)	1.68 (0.95–2.53)	38.91 (19.97–61.86)	34.95 (18–56.69)	−0.1 (−0.32 to 0.16)
MASLD						
Female	4,186.46 (3,068.87–5,468.37)	8,004.74 (5,930.47–10,798.27)	0.91 (0.37–1.77)	1.03 (0.75–1.33)	0.73 (0.54–0.98)	−0.28 (−0.48 to 0.03)

TPC, total percentage change; UI, uncertainty interval; T2DM, type 2 diabetes mellitus; MASLD, metabolic dysfunction-associated steatotic liver disease.

### 3.2 T2DM burden

**Figure 3:**
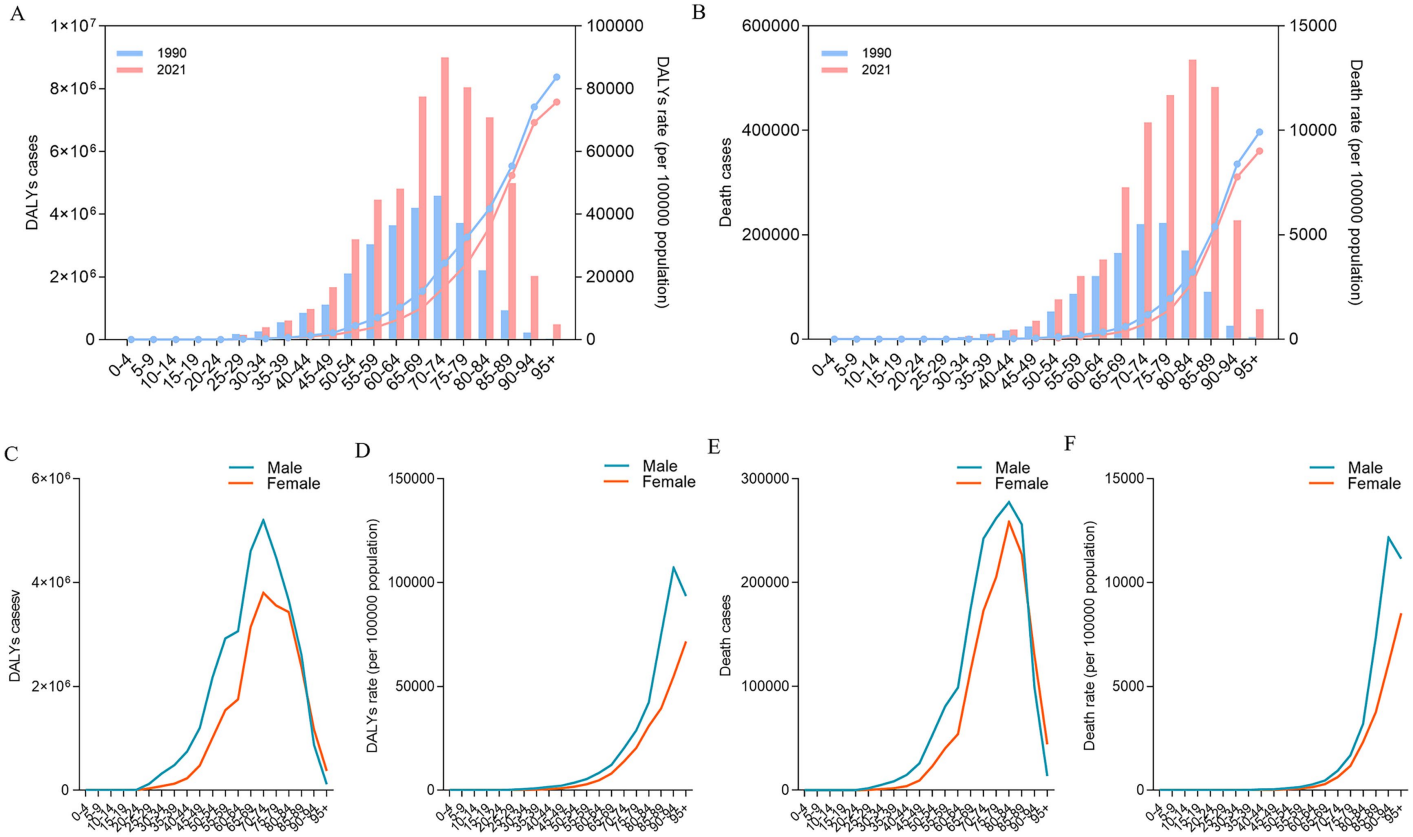
Burden of T2DM in Mainland China by different age groups. **(A)** DALYs cases and DALYs rate in 1990 and 2021; **(B)** Death cases and death rate in 1990 and 2021; **(C)** DALYs cases by sex in 2021; **(D)** DALYs rate by sex in 2021; **(E)** Death cases by sex in 2021; **(F)** Death rate by sex in 2021.

In 2021, the total age-standardized DALYs rate caused by T2DM was 569.84 (95% UI: 435.43–734.18), and the total age-standardized deaths rate was 8.74 (95% UI: 7.26–10.35; Tables 1–6). From 1990 to 2021, the numbers of DALYs and deaths, age-standardized DALYs rate caused by T2DM all showed increasing trends, with corresponding TPCs of 1.9, 1.63, and 0.3, respectively, and the percentage increase in males was higher than that in females during 1990–2021 (Figure 2A–C, Tables 1–6). For DALYs, the estimated number in males reached 6.04 million (95% UI: 4.64–7.84) in 2021, with a TPC of 2.14, while the corresponding number in females reached 5.42 million (95% UI: 4.19–6.95) in 2021, with a TPC of 1.66. For age-standardized DALYs rate, the estimated rate in males reached 620.06 (95% UI: 473.83–801.09) in 2021, with a TPC of 0.44, while corresponding rate in females reached 523.52 (95% UI: 401.27–676.86) in 2021, with a TPC of 0.16. For deaths cases, the estimated number in males reached 86, 026.3 (95% UI: 66, 966.42–108, 719.31) in 2021, with a TPC of 2.07, while the corresponding number in females reached 88, 489.08 (95% UI: 70, 122.5–109, 406.66) in 2021, with a TPC of 1.3 (Tables 1–6). In addition, the DALYs and deaths cases in each age group in 2021 were all higher than those in 1990 (Figures 3A,B). Although the total age-standardized deaths rate showed a slight decrease from 1990 to 2021, with a TPC of −0.06, the rate in males still reached 9.97 (95% UI: 7.83–12.45) in 2021, with a TPC of 0.1, while that in females decreased to 8.08 (95% UI: 6.42–9.95) in 2021, with a TPC of −0.18 (Figure 2D, Tables 1–6). In summary, the burden of T2DM showed an increasing trend from 1990 to 2021, and that both the absolute burden and the growth rate of T2DM were higher in men than in women.

The results of the age effect analysis indicated that the risk of T2DM in the overall population initially increased and then decreased with age in 2021 (Figures 3A–C,E). The highest number of DALYs was in the 65–69 years age group and for deaths was in the 70–74 years age group, for both males and females (Figures 3C,E). In males, the highest risks for DALYs rate and deaths rate were both in those aged 90–94 years (Figures 3D,F). In females, the highest risk for DALYs rate was in those aged 75–79 years, and for deaths rate was in those aged 95+ years (Figures 3D,F).

### 3.3 Hypertension burden

**Figure 4:**
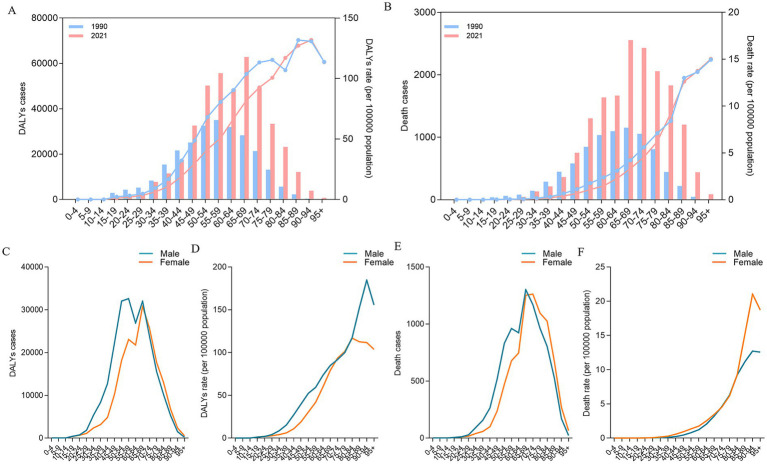
Burden of hypertension in Mainland China by different age groups. **(A)** DALYs cases and DALYs rate in 1990 and 2021; **(B)** Death cases and death rate in 1990 and 2021; **(C)** DALYs cases by sex in 2021; **(D)** DALYs rate by sex in 2021; **(E)** Death cases by sex in 2021; **(F)** Death rate by sex in 2021.

In 2021, the total age-standardized DALYs rate caused by hypertension was 2,788.47 (95% UI: 2,170.34–3,503.6), and the total age-standardized deaths rate was 158.01 (95% UI: 122.81–198.51; Tables 1–6). From 1990 to 2021, the numbers of DALYs and deaths caused by hypertension showed increasing trends, with TPCs of 1.01 and 1.38, respectively, and the percentage increase in males was higher than those in females during 1990–2021 (Figure 2A,C; Tables 1–6). For DALYs, the estimated number in males reached 32.59 million (95% UI: 23.72–43.62) in 2021, with a TPC of 1.22, while the corresponding number in females reached 23.10 million (95% UI: 17.25–30.78) in 2021, with a TPC of 0.77. For deaths cases, the estimated number in males reached 1.61 million (95% UI: 1.19–2.13) in 2021, with a TPC of 1.65, while the corresponding number in females reached 1.29 million (95% UI: 0.96–1.74) in 2021, with a TPC of 1.11 (Tables 1–6). In addition, the DALYs and deaths cases in each age group in 2021 were all higher than those in 1990 (Figures 4A,B). Although the total age-standardized DALYs rate and age-standardized deaths rate both showed a decreasing trend from 1990 to 2021, the two indicators still remained highest among the five metabolic diseases (Figures 2B,D). For age-standardized DALYs rate, the estimated rate in males was 3,582.86 (95% UI: 2,653.82–4,720.82) in 2021, with a TPC of −0.14, and the corresponding rate in females was 2,142.69 (95% UI: 1,581.73–2,850.76) in 2021, with a TPC of −0.37. For age-standardized deaths rate, the estimated rate in males was 207.32 (95% UI: 154.26–267.99) in 2021, with a TPC of −0.09, while corresponding rate in females was 124.35(95% UI: 93.01–167.13) in 2021, with a TPC of −0.34 (Tables 1–6). Altogether, the absolute burden of hypertension showed an increasing trend, and the age-standardized DALYs and deaths rates showed decreased trends from 1990 to 2021, with women experiencing a greater decline than men.

The results of the age effect analysis indicated that the absolute burden of hypertension in the overall population initially increased and then decreased with age in 2021 (Figures 4A,B). The highest number of DALYs was in the 70–74 years age group and for deaths cases was in the 80–84 years age group for both males and females (Figures 4C,E). The DALYs rate and deaths rate showed upward trends with age in both males and females in 2021 (Figures 4A,B). The highest risks for DALYs rate and deaths rate in males were in those aged 90–94 years, and in females were in those aged 95+ years (Figures 4D,F).

### 3.4 Obesity burden

**Figure 5:**
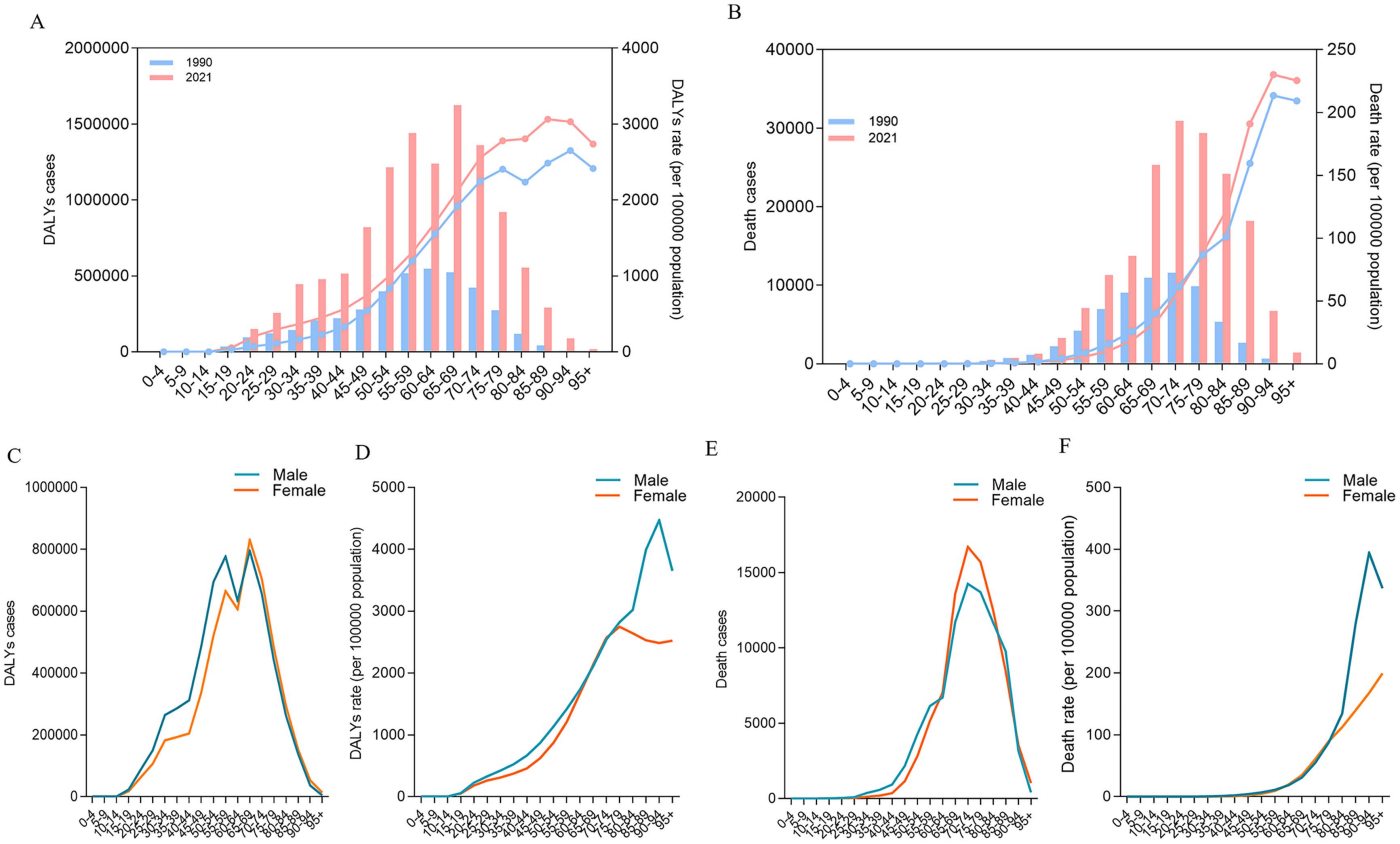
Burden of obesity in Mainland China by different age groups. **(A)** DALYs cases and DALYs rate in 1990 and 2021; **(B)** Death cases and death rate in 1990 and 2021; **(C)** DALYs cases by sex in 2021; **(D)** DALYs rate by sex in 2021; **(E)** Death cases by sex in 2021; **(F)** Death rate by sex in 2021.

In 2021, the total age-standardized DALYs rate caused by obesity was 1,028.85 (95% UI: 428.39–1,635.04), and the total age-standardized deaths rate was 30.01 (95% UI: 15.41–47.75; Tables 1–6). From 1990 to 2021, the numbers of DALYs and deaths, age-standardized DALYs rate and age-standardized deaths rate caused by obesity all showed increasing trends, with TPCs of 2.84, 3.05, 0.65, and 0.43, respectively, and the percentage increases in males were all higher than that in females during 1990–2021 (Figures 2A–D; Tables 1–6). For DALYs, the estimated number in males reached 10.38 million (95% UI: 4.76–17.05) in 2021, with a TPC of 3.04, while corresponding number in females reached 10.49 million (95% UI: 4.06–17.01) in 2021, with a TPC of 2.66. For deaths cases, the estimated number in males reached 0.28 million (95% UI: 0.14–0.47) in 2021, with a TPC of 3.38, while in females reached 0.29 million (95% UI: 0.14–0.49) in 2021, with a TPC of 2.78 (Tables 1–6). For age-standardized DALYs rate, the estimated rate in males reached 1,079.27 (95% UI: 508.29–1,769.82) in 2021, with a TPC of 0.8, and in females reached 984.13 (95% UI: 386.69–1,579.28) in 2021, with a TPC of 0.51. For age-standardized deaths rate, the estimated rate in males reached 33.6 (95% UI: 17.67–55.75) in 2021, with a TPC of 0.59, and in females reached 27.63 (95% UI: 13.2–46.67) in 2021, with a TPC of 0.3 (Tables 1–6). In addition, the DALYs and deaths cases in each age group in 2021 were all higher than those in 1990 (Figures 5A,B). Collectively, the burden of obesity showed an increasing trend from 1990 to 2021, and the growth rate of obesity was higher in men than in women.

The results of the age effect analysis indicated that the absolute burden of obesity in the overall population initially increased and then decreased with age in 2021 (Figures 5A,B). The highest numbers of DALYs and deaths were both in the 70–74 years age group for both males and females (Figures 5C,E). The DALYs rate and deaths rate showed upward trends with age in both males and females in 2021 (Figures 5A,B). The highest risks for DALYs rate and deaths rate in males were in those aged 90–94 years and 95+ years, respectively. For females, both indicators peaked in the 95+ years age group (Figures 5D,F).

### 3.5 Hypercholesterolemia burden

In 2021, the total age-standardized DALYs rate caused by hypercholesterolemia was 920.69 (95% UI: 511.71–1359.77), and the total age-standardized deaths rate was 44.97 (95% UI: 23.22–69.89; Tables 1–6). From 1990 to 2021, the numbers of DALYs and deaths, as well as age-standardized deaths rate caused by hypercholesterolemia, all showed increasing trends, with TPCs of 1.33, 1.94 and 0.04, respectively, and the percentage increases in males were all higher than those in females (Figure 2A,C,D; Tables 1–6). For DALYs, the estimated number in males reached 11.17 million (95% UI: 6.06–16.72) in 2021, with a TPC of 1.55, while that in females reached 7.24 million (95% UI: 3.91–11.21), with a TPC of 1.05. For deaths cases, the estimated number in males reached 0.47 million (95% UI: 232, 649.94–732, 842.74) in 2021, with a TPC of 2.18, while the number in females reached 0.36 million (95% UI: 0.18–0.58), with a TPC of 1.68. For age-standardized deaths rate, the estimated rate in males reached 58.57 (95% UI: 28.56–90.82) in 2021, with a TPC of 0.2, while that in females was 34.95 (95% UI: 18–56.69), with a TPC of −0.1 (Tables 1–6). Although the total age-standardized DALYs rate showed a slight decrease from 1990 to 2021, with a TPC of −0.03, the rate in males still reached 1193.16 (95% UI: 635.37–1,787.06) in 2021, with a TPC of 0.11, while that in females was 679.55 (95% UI: 369.17–1,050.9), with a TPC of −0.2 (Figure 2B, Tables 1–6). Based on these results, the burden of hypercholesterolemia showed an increasing trend from 1990 to 2021, and both the absolute burden and the growth rate of hypercholesterolemia were higher in men than in women.

**Figure 6:**
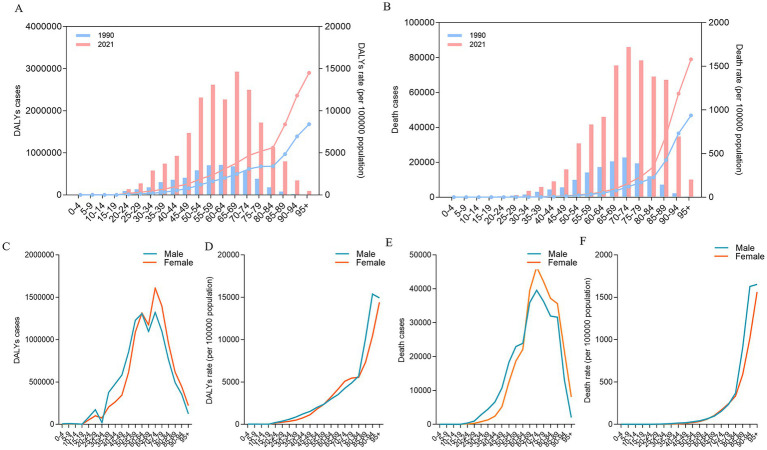
Burden of hypercholesterolemia in Mainland China by different age groups. **(A)** DALYs cases and DALYs rate in 1990 and 2021; **(B)** Death cases and death rate in 1990 and 2021; **(C)** DALYs cases by sex in 2021; **(D)** DALYs rate by sex in 2021; **(E)** Death cases by sex in 2021; **(F)** Death rate by sex in 2021.

The results of the age effect analysis indicated that the absolute burden of hypercholesterolemia in the overall population initially increased and then decreased with age in 2021 (Figures 6A,B). The highest numbers of DALYs were 65–69 years and 70–74 years age groups for males and females, respectively (Figure 6C). The highest numbers of deaths were in the group aged 80–84 years for both males and females (Figure 6E). In males, the highest risk for DALYs rate was those aged 90–94 years, and for deaths rate was those aged 95+ years (Figures 6D,F). In females, the highest risks for DALYs rate and deaths rate were both in those aged 95+ years (Figures 6D,F).

### 3.6 MASLD burden

In 2021, the total age-standardized DALYs rate caused by MASLD was 19.83 (95% UI: 15.29–24.98), and the total age-standardized deaths rate was 0.82 (95% UI: 0.64–1.03; Tables 1–6). From 1990 to 2021, the numbers of DALYs and deaths caused by MASLD showed increasing trends, with TPCs of 0.64 and 1.01, respectively, and the percentage increases in males were higher than those in females (Tables 1–6). For DALYs, the estimated number in males reached 0.23 million (95% UI: 0.16–0.32) in 2021, with a TPC of 0.72, while the corresponding number in females reached 0.18 million (95% UI: 0.14–0.25), with a TPC of 0.54. For deaths cases, the estimated number in males reached 8,743.63 (95% UI: 6,203.62–11,893.48) in 2021, with a TPC of 1.1, while that in females reached 8,004.74 (95% UI: 5,930.47–10,798.27), with a TPC of 0.91 (Tables 1–6). For age-standardized DALYs rate, the estimated rate in males decreased to 22.93 (95% UI: 16.59–31.03) in 2021, with a TPC of −0.19, and that in females decreased to 16.81 (95% UI: 12.56–22.53), with a TPC of −0.35. For age-standardized deaths rate, the estimated rate in males decreased to 0.92 (95% UI: 0.67–1.24) in 2021, with a TPC of −0.11, while that in females decreased to 0.73 (95% UI: 0.54–0.98), with a TPC of −0.28 (Tables 1–6). Taken together, the absolute burden of MASLD showed an increasing trend, and the age-standardized DALYs and deaths rates showed decreased trends from 1990 to 2021, with women experiencing a greater decline than men.

**Figure 7:**
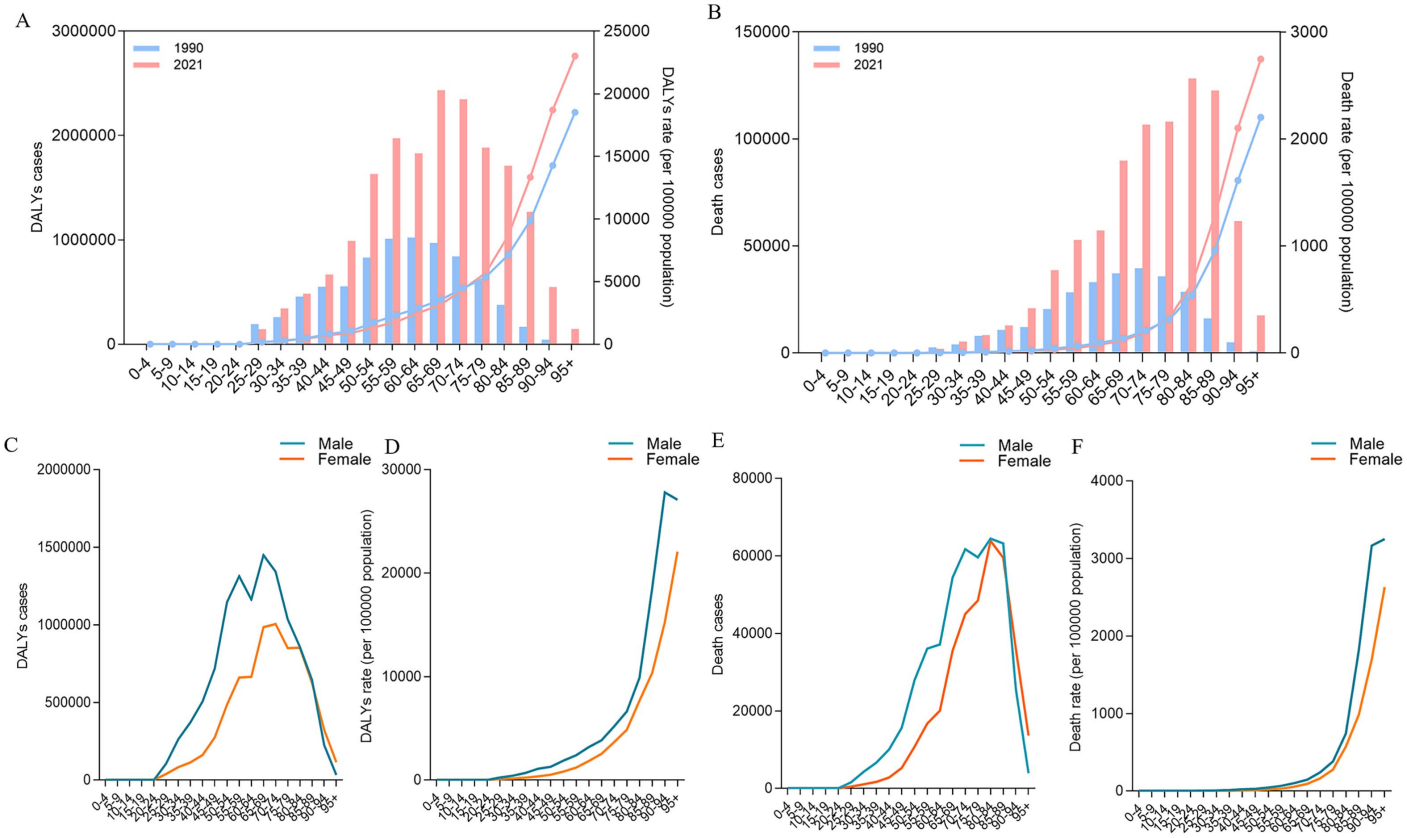
Burden of MASLD in Mainland China by different age groups. **(A)** DALYs cases and DALYs rate in 1990 and 2021; **(B)** Death cases and death rate in 1990 and 2021; **(C)** DALYs cases by sex in 2021; **(D)** DALYs rate by sex in 2021; **(E)** Death cases by sex in 2021; **(F)** Death rate by sex in 2021.

The results of the age effect analysis indicated that the absolute burden of MASLD in the overall population initially increased and then decreased with age in 2021 (Figures 7A,B). The highest numbers of DALYs were in the 55–59 years and 65–69 years age groups for males and females, respectively (Figure 7C). For deaths, the highest number was in the group aged 65–69 years for males and the group aged 70–74 years for females (Figure 7E). The highest DALYs rate was in the 90–94 years age group for males and the 80–84 years age group for females, and the highest risk for deaths rate was in those aged 90–94 years for both males and females (Figures 7D,F).

## 4 Discussion

In previous GBD studies, the burden of mortality and DALYs was assessed in isolation for each individual disease. However, metabolic diseases do not exist as distinct entities, but often coexist and share certain underlying mechanisms, which collectively contribute to disability, cancers, cardiovascular diseases, and premature death (10). According to GBD 2021 estimates, we illustrated the health burden of five common metabolic diseases in Mainland China, namely T2DM, hypertension, obesity, hypercholesterolemia, and MASLD, across temporal, sex, and age dimensions, using the indicator of disability-adjusted life years. Since only T2DM and MASLD are classified as diseases in the GBD framework (hypertension, obesity, and hypercholesterolemia are listed as risk factors), prevalence was not the primary focus of our analysis. The national burden of these common metabolic diseases has increased over the past three decades, with relative increases ranging from 0.64-fold to 2.84-fold. Currently, the greatest burden of metabolic diseases in Mainland China is caused by hypertension, and obesity showed the fastest percentage increase in its burden.

In 2021, the highest number of DALYs and age-standardized DALYs rate in Mainland China were observed in hypertension, followed by obesity, hypercholesterolemia, T2DM and MASLD. The highest number of deaths and age-standardized deaths rate were also observed in hypertension, followed by hypercholesterolemia, obesity, T2DM and MASLD. Regarding temporal distribution, we found that the numbers of DALYs and deaths for the five common metabolic diseases increased from 1990 to 2021 in Mainland China, a finding consistent with previous studies (1, 2, 11). The steady increase in the burden of non-communicable diseases may be attributed to multiple factors, including environmental pollution, changes in lifestyle patterns, urbanization, population aging, and even the effects of COVID-19 (12, 13). The TPC in age-standardized DALYs rate for T2DM and obesity increased from 1990 to 2021, as did age-standardized deaths rate for obesity and hypercholesterolemia. Therefore, it can be concluded that obesity has become a major national health threat, a trend consistent with global GBD studies (1, 14). It has been well documented in the literature that obesity increases health risks. For example, it contributes to the risk of mental disturbance (15), behavioral issues (16) and premature mortality (17, 18). In addition, obesity can alter glucose metabolism and insulin resistance, both of which increase the risk of other chronic metabolic diseases (19). Therefore, education on the importance of physical activity, nutrition and chronic disease management is urgently needed to mitigate the burden of obesity and other related chronic metabolic diseases.

Regarding sex distribution, for almost all five common metabolic diseases, the burden of DALYs and deaths was heavier in males than in females. There are several possible reasons for this phenomenon. Firstly, women are more sensitive to health information, more aware of their risk factors, and more capable of seeking medical assistance and implementing primary prevention measures compared to men (20, 21). Secondly, men tend to prefer high-calorie, high-fat, and high-sugar foods, while women tend to stick to healthy diets (22–24). Thirdly, estrogen exerts a protective effect against oxidative stress, a mechanism that is less pronounced in males (25). These findings indicate that gender differences in DALYs and deaths burden are likely driven by a combination of genetic, lifestyle, and environmental factors. Regarding age distribution, the greatest burden of metabolic conditions was observed in 55–74 years age group, a pattern largely attributable to population aging and population growth (26). Therefore, targeted health education and intervention for specific age groups is essential for reducing the burden of metabolic diseases. To address the growing health and economic burden of metabolic diseases in Mainland China, the following targeted interventions may be effective. Firstly, early intervention and education programs for common metabolic diseases need to be significantly scaled up, with a strong focus on early diagnosis and treatment in high-risk populations. Secondly, disease care in hospitals should be combined with prevention and treatment in the community. Through the integration of these models, patients will be able to access treatment more easily and will adhere to their treatment better. In addition, patients of chronic metabolic diseases will receive better follow-up on their pretreatment monitoring. Thirdly, governmental bodies and institutions should work together to improve access to essential diabetes medications, reduce drug costs, and enhance the affordability of health insurance for rural residents.

There are, however, some limitations in our research. Firstly, China is a large country, and the disease burden varies greatly from province to province, which complicates the assessment of disease burden in specific provinces. Secondly, this study may have limitations due to the inherent methodology of GBD. For example, the limited availability of national registry data and a large number of undiagnosed cases limit the accuracy of the GBD database. Thirdly, as mentioned in the methods section, hypertension, obesity, and hypercholesterolemia are categorized as risk factors (high systolic blood pressure, high body-mass index, and high LDL cholesterol, respectively) rather than distinct diseases in the GBD framework. This categorization may to some extent underestimate the true burden of these metabolic conditions.

It is noteworthy that China ranks first globally in both MASLD prevalent cases and annual incident cases (27), but its attributable DALYs and deaths were substantially lower than obesity and T2DM. This paradox reflects GBD methodology: the burden estimation primarily captures advanced liver disease (cirrhosis, hepatocellular carcinoma), while early-stage steatosis lacks sufficient disability weights for DALY incorporation. Moreover, MASLD patients typically die from cardiovascular events rather than liver disease itself, and MASLD is rarely certified as the direct cause of death. Consequently, the reported MASLD burden represents a conservative estimate of its advanced hepatic sequelae.

In conclusion, there has been a significant increase in the burden of metabolic diseases over the past three decades although there are differences in health burdens, which imposes significant health challenges in Mainland China. Attention is needed to address the concerning surge in the DALYs and mortality associated with these metabolic conditions as well as sex disparities of burden from metabolic diseases. Implementing effective preventive and therapeutic strategies at the individual, communal, and national levels remains urgent.

## Data Availability

Publicly available datasets were analyzed in this study. This data can be found at: https://ghdx.healthdata.org/gbd-2021.

## Footnotes

1
https://ghdx.healthdata.org/gbd-2021
