# Supplementary material for: Burden of metabolic diseases in mainland China from 1990 to 2021: results from the global burden of disease study 2021
Source: Front Public Health. 2026 Jul 14;14:1846594. doi: 10.3389/fpubh.2026.1846594 (PMC13407768; doi:10.3389/fpubh.2026.1846594)
Supplement: Supplementary file 1 [file Data_Sheet_1.doc]

**Supplementary Table1 The burden of DALYs and deaths of patients with T2DM in 2021 in China, with percentage changes from 1990 to 2021**

| Age | DALYs (95% UI) | | |  | Mortality (95% UI) | | |
| --- | --- | --- | --- | --- | --- | --- | --- |
|  | Number | Rate | TPC (1990-2021) |  | Number | Rate | TPC (1990-2021) |
| ＜5 | - | - | - |  | - | - | - |
| 5-9 | - | - | - |  | - | - | - |
| 10-14 | - | - | - |  | - | - | - |
| 15-19 | 40464.84 (24700.09-62750.68) | 54.19 (33.08-84.03) | 0.93 (0.6-1.29) |  | 28.18 (22.63-35.55) | 0.04 (0.03-0.05) | -0.39 (-0.52--0.11) |
| 20-24 | 150270.21 (90928.06-223553.02) | 205.36 (124.26-305.51) | 1.83 (1.43-2.34) |  | 83.26 (66.98-104.35) | 0.11 (0.09-0.14) | -0.09 (-0.33-0.35) |
| 25-29 | 257069.18 (159425.67-372576.57) | 297.25 (184.35-430.81) | 1.67 (1.38-2.06) |  | 142.31 (114.76-179.38) | 0.16 (0.13-0.21) | 0.1 (-0.16-0.54) |
| 30-34 | 445555.85 (287547.97-644230.13) | 367.76 (237.34-531.75) | 1.27 (1.05-1.52) |  | 496.87 (409.34-618.88) | 0.41 (0.34-0.51) | -0.05 (-0.27-0.28) |
| 35-39 | 478701.48 (321212.78-677137.76) | 451.76 (303.14-639.03) | 0.98 (0.8-1.2) |  | 772 (632.58-943.07) | 0.73 (0.6-0.89) | -0.11 (-0.31-0.18) |
| 40-44 | 516024.64 (358919.4-711446.2) | 563.75 (392.12-777.25) | 0.71 (0.54-0.89) |  | 1299.69 (1044.31-1608.27) | 1.42 (1.14-1.76) | -0.17 (-0.36-0.11) |
| 45-49 | 821353.44 (586240.02-1093457.55) | 744.51 (531.39-991.15) | 0.38 (0.23-0.53) |  | 3313.57 (2625.73-4124.91) | 3 (2.38-3.74) | -0.31 (-0.48--0.08) |
| 50-54 | 1214263.34 (901260.31-1586242.67) | 1004.69 (745.71-1312.47) | 0.2 (0.07-0.34) |  | 7092.71 (5614.8-8730.49) | 5.87 (4.65-7.22) | -0.34 (-0.5--0.1) |
| 55-59 | 1441336.41 (1091872.45-1870582.35) | 1310.99 (993.13-1701.42) | 0.1 (-0.03-0.22) |  | 11315.17 (9125.8-13935.33) | 10.29 (8.3-12.68) | -0.36 (-0.52--0.15) |
| 60-64 | 1239180.64 (963043.82-1584766.36) | 1697.39 (1319.14-2170.76) | 0.09 (-0.04-0.23) |  | 13752.35 (11087.62-16674.05) | 18.84 (15.19-22.84) | -0.27 (-0.43--0.02) |
| 65-69 | 1625129.09 (1278589.56-2043646.08) | 2118.72 (1666.92-2664.34) | 0.1 (-0.02-0.24) |  | 25311.99 (20906.85-30639.51) | 33 (27.26-39.95) | -0.18 (-0.37-0.06) |
| 70-74 | 1361304.41 (1109628.72-1669511.52) | 2554.21 (2081.99-3132.5) | 0.14 (0.01-0.29) |  | 30945.99 (25675.33-37248.33) | 58.06 (48.17-69.89) | -0.06 (-0.25-0.2) |
| 75-79 | 920802.99 (760301.17-1108074.03) | 2780.29 (2295.67-3345.74) | 0.16 (0.02-0.33) |  | 29392.71 (24495.73-35123.73) | 88.75 (73.96-106.05) | 0.02 (-0.2-0.31) |
| 80-84 | 555300.42 (464979.13-659273.75) | 2805.7 (2349.35-3331.04) | 0.25 (0.11-0.42) |  | 24184.9 (19857.27-28732.32) | 122.2 (100.33-145.17) | 0.21 (-0.02-0.5) |
| 85-89 | 291904.85 (247769.72-340362.52) | 3064.38 (2601.05-3573.08) | 0.23 (0.1-0.39) |  | 18195.64 (14946.91-21584.6) | 191.02 (156.91-226.59) | 0.2 (-0.02-0.45) |
| 90-94 | 88862.26 (74514.55-104428.2) | 3030.79 (2541.43-3561.69) | 0.14 (0.02-0.29) |  | 6747.57 (5411.12-7974.13) | 230.14 (184.55-271.97) | 0.08 (-0.11-0.28) |
| 95+ | 17493.84 (13919.72-20831.32) | 2737.27 (2178.02-3259.48) | 0.13 (-0.01-0.28) |  | 1440.48 (1078.75-1746.46) | 225.39 (168.79-273.27) | 0.08 (-0.13-0.28) |

DALYs, disability-adjusted life years; TPC, total percentage change

Supplementary Table 2 The burden of DALYs and death of patients with hypertension in 2021 in China, with percentage changes from 1990 to 2021

| Age | DALYs (95% UI) | | |  | Mortality (95% UI) | | |
| --- | --- | --- | --- | --- | --- | --- | --- |
|  | Number | Rate | TPC (1990-2021) |  | Number | Rate | TPC (1990-2021) |
| ＜5 | - | - | - |  | - | - | - |
| 5-9 | - | - | - |  | - | - | - |
| 10-14 | - | - | - |  | - | - | - |
| 15-19 | 4131.73 (2877-5746.31) | 5.53 (3.85-7.7) | -0.67 (-0.77--0.35) |  | 53.88 (36.21-75.58) | 0.07 (0.05-0.1) | -0.69 (-0.79--0.37) |
| 20-24 | 7390.42 (5061.95-9755.14) | 10.1 (6.92-13.33) | -0.55 (-0.68--0.22) |  | 101.02 (67.63-136.8) | 0.14 (0.09-0.19) | -0.58 (-0.7--0.23) |
| 25-29 | 152111.55 (50920.67-257862.82) | 175.89 (58.88-298.17) | 0.09 (-0.58-1.86) |  | 2079.88 (686.22-3492.66) | 2.4 (0.79-4.04) | 0.06 (-0.59-1.79) |
| 30-34 | 397908.2 (158390.8-630545.82) | 328.43 (130.74-520.45) | 0.11 (-0.51-1.87) |  | 6101.02 (2355.97-9757.97) | 5.04 (1.94-8.05) | 0.08 (-0.55-1.75) |
| 35-39 | 607782.91 (258295.89-951054.76) | 573.58 (243.76-897.53) | -0.05 (-0.56-1.09) |  | 10366.78 (4405.7-16335.59) | 9.78 (4.16-15.42) | -0.08 (-0.58-1.03) |
| 40-44 | 975249.31 (504185.55-1505829.2) | 1065.45 (550.82-1645.11) | -0.16 (-0.58-0.88) |  | 18687.09 (9569.47-28924.07) | 20.42 (10.45-31.6) | -0.19 (-0.59-0.81) |
| 45-49 | 1674890.63 (948386.09-2481523.55) | 1518.19 (859.66-2249.36) | -0.3 (-0.6-0.21) |  | 35368.57 (19741.51-53170.18) | 32.06 (17.89-48.2) | -0.33 (-0.62-0.17) |
| 50-54 | 3195975.14 (1980315.38-4612942.3) | 2644.38 (1638.53-3816.8) | -0.4 (-0.66-0.06) |  | 76444.26 (47220.81-112085.62) | 63.25 (39.07-92.74) | -0.43 (-0.68-0.02) |
| 55-59 | 4464276.98 (2765201.61-6145542.82) | 4060.56 (2515.14-5589.79) | -0.42 (-0.64--0.03) |  | 121115.21 (73461.22-167604.74) | 110.16 (66.82-152.45) | -0.45 (-0.66--0.08) |
| 60-64 | 4810296.07 (3003213.86-6550674.56) | 6588.98 (4113.7-8972.89) | -0.36 (-0.58-0.02) |  | 152752.02 (94378.09-209367.78) | 209.23 (129.28-286.78) | -0.39 (-0.6--0.02) |
| 65-69 | 7754162.96 (5126252.33-10421680.75) | 10109.27 (6683.2-13586.97) | -0.34 (-0.55-0.05) |  | 291436.46 (191166.45-390456.76) | 379.95 (249.23-509.05) | -0.37 (-0.58-0.01) |
| 70-74 | 9000153.98 (6282540.31-11678724.17) | 16886.97 (11787.92-21912.77) | -0.31 (-0.53--0.01) |  | 415278.24 (288905.01-543951.32) | 779.19 (542.07-1020.61) | -0.33 (-0.56--0.04) |
| 75-79 | 8049314.64 (5761531.64-10635026.14) | 24304.27 (17396.49-32111.62) | -0.26 (-0.49-0.07) |  | 467556.21 (334458.55-626318.73) | 1411.75 (1009.87-1891.12) | -0.28 (-0.5-0.04) |
| 80-84 | 7088053.24 (5015041.53-9344500.73) | 35812.98 (25338.91-47213.87) | -0.14 (-0.41-0.28) |  | 535752.8 (377619.45-710569.73) | 2706.94 (1907.95-3590.21) | -0.16 (-0.42-0.26) |
| 85-89 | 4988861.78 (3570584.5-6588186.91) | 52372.37 (37483.5-69161.86) | -0.05 (-0.34-0.39) |  | 483240.99 (343885.32-642456.83) | 5073 (3610.06-6744.42) | -0.06 (-0.34-0.38) |
| 90-94 | 2030446.28 (1316369.48-2779145.2) | 69251.53 (44896.83-94787.08) | -0.07 (-0.39-0.49) |  | 227758.12 (147410.78-311862.66) | 7768.05 (5027.67-10636.56) | -0.07 (-0.39-0.48) |
| 95+ | 484403.92 (293982.36-689666.49) | 75794.88 (45999.54-107912.4) | -0.1 (-0.47-0.47) |  | 57624.13 (34922.3-81703.73) | 9016.47 (5464.31-12784.22) | -0.09 (-0.46-0.47) |

DALYs, disability-adjusted life years; TPC, total percentage change

Supplementary Table 3 The burden of DALYs and death of patients with obesity in 2021 in China, with percentage changes from 1990 to 2021

| Age | DALYs (95% UI) | | |  | Mortality (95% UI) | | |
| --- | --- | --- | --- | --- | --- | --- | --- |
|  | Number | Rate | TPC (1990-2021) |  | Number | Rate | TPC (1990-2021) |
| ＜5 | 8001.9 (2880.03-16197.92) | 10.3 (3.71-20.86) | 0.64 (0.19-1.01) |  | 0.44 (0.18-0.8) | 0 (0-0) | -0.97 (-0.98--0.92) |
| 5-9 | 8483.92 (3448.79-16656.95) | 9.84 (4-19.33) | 0.99 (0.69-1.28) |  | 1.94 (0.95-3.19) | 0 (0-0) | -0.78 (-0.84--0.64) |
| 10-14 | 3741.41 (1499.25-7070.45) | 5.01 (2.01-9.47) | 1.16 (0.83-1.49) |  | 2.2 (1.08-3.67) | 0 (0-0) | -0.5 (-0.66--0.25) |
| 15-19 | 136067.88 (62968.99-213609.75) | 185.95 (86.05-291.92) | 1.65 (0.95-2.44) |  | 498.66 (272.97-752.04) | 0.68 (0.37-1.03) | 0.56 (-0.05-1.27) |
| 20-24 | 274029.9 (128841.56-419419.35) | 316.86 (148.98-484.98) | 1.59 (1.03-2.1) |  | 1206.1 (605.55-1881.02) | 1.39 (0.7-2.18) | 0.68 (0.07-1.26) |
| 25-29 | 580209.06 (264060.91-871763.1) | 478.9 (217.96-719.55) | 1.35 (0.9-1.73) |  | 3624.58 (1686.16-5864.1) | 2.99 (1.39-4.84) | 0.74 (0.18-1.25) |
| 30-34 | 743817.08 (334852.85-1180105.57) | 701.96 (316.01-1113.69) | 1.12 (0.7-1.47) |  | 5850.44 (2577.76-9812.42) | 5.52 (2.43-9.26) | 0.64 (0.09-1.15) |
| 35-39 | 926829.05 (391886.91-1510936.28) | 1012.56 (428.13-1650.69) | 0.89 (0.45-1.25) |  | 9042.9 (3939.86-14987.7) | 9.88 (4.3-16.37) | 0.5 (-0.02-1.02) |
| 40-44 | 1473161.69 (606260.41-2381994.87) | 1335.33 (549.54-2159.14) | 0.69 (0.32-1) |  | 15909.2 (7082.41-27340.96) | 14.42 (6.42-24.78) | 0.32 (-0.13-0.8) |
| 45-49 | 13176.82 (5227.74-26641.2) | 13.76 (5.46-27.82) | 0.91 (0.59-1.2) |  | 1.52 (0.72-2.64) | 0 (0-0) | -0.86 (-0.9--0.72) |
| 50-54 | 2314683.46 (873005.81-3826385.14) | 1915.19 (722.33-3165.99) | 0.56 (0.09-0.88) |  | 30824.6 (13355.26-51664.31) | 25.5 (11.05-42.75) | 0.23 (-0.2-0.66) |
| 55-59 | 2619385.08 (979977.52-4366628.12) | 2382.51 (891.36-3971.74) | 0.47 (0.01-0.8) |  | 41633.01 (17847.96-70341.92) | 37.87 (16.23-63.98) | 0.16 (-0.27-0.6) |
| 60-64 | 2269705.85 (880613.96-3745548.31) | 3108.97 (1206.24-5130.52) | 0.54 (-0.01-0.92) |  | 46033.45 (21203.66-77715.12) | 63.06 (29.04-106.45) | 0.29 (-0.21-0.77) |
| 65-69 | 2930209.09 (1146811.74-4838145.24) | 3820.18 (1495.12-6307.59) | 0.53 (0.01-0.98) |  | 75452.4 (33418.47-124905.65) | 98.37 (43.57-162.84) | 0.31 (-0.17-0.81) |
| 70-74 | 2497829.45 (1041131.13-4143303.19) | 4686.67 (1953.47-7774.07) | 0.51 (-0.05-0.98) |  | 85978.57 (40262.36-138806.61) | 161.32 (75.54-260.44) | 0.33 (-0.18-0.86) |
| 75-79 | 1719293.49 (755408.75-2828535.02) | 5191.27 (2280.9-8540.54) | 0.54 (-0.09-1.08) |  | 78356.65 (37884.26-124120.77) | 236.59 (114.39-374.77) | 0.39 (-0.17-1.03) |
| 80-84 | 1109721.1 (497471.75-1807463.28) | 5606.96 (2513.52-9132.36) | 0.65 (-0.05-1.53) |  | 69138.52 (34713.8-111757.98) | 349.33 (175.39-564.67) | 0.54 (-0.09-1.57) |
| 85-89 | 798200.08 (369680.78-1282133.68) | 8379.39 (3880.86-13459.66) | 0.73 (0.01-1.81) |  | 67207.13 (32965.82-109186.98) | 705.53 (346.07-1146.23) | 0.66 (-0.01-1.86) |
| 90-94 | 345958.96 (167165.07-563650) | 11799.47 (5701.42-19224.16) | 0.7 (0.01-1.81) |  | 34769.04 (17230.92-56212.67) | 1185.85 (587.69-1917.22) | 0.62 (-0.01-1.79) |
| 95+ | 92666.33 (43781.33-161739.3) | 14499.54 (6850.48-25307.42) | 0.73 (0-1.79) |  | 10093.1 (4882.14-17188.31) | 1579.27 (763.91-2689.46) | 0.69 (-0.01-1.79) |

DALYs, disability-adjusted life years; TPC, total percentage change

Supplementary Table 4 The burden of DALYs and death of patients with hypercholesterolemia in 2021 in China, with percentage changes from 1990 to 2021

| Age | DALYs (95% UI) | | |  | Mortality (95% UI) | | |
| --- | --- | --- | --- | --- | --- | --- | --- |
|  | Number | Rate | TPC (1990-2021) |  | Number | Rate | TPC (1990-2021) |
| ＜5 | - | - | - |  | - | - | - |
| 5-9 | - | - | - |  | - | - | - |
| 10-14 | - | - | - |  | - | - | - |
| 15-19 | - | - | - |  | - | - | - |
| 20-24 | - | - | - |  | - | - | - |
| 25-29 | 144435.38 (100507.9-188072.88) | 167.01 (116.22-217.47) | -0.05 (-0.21-0.16) |  | 1988.15 (1413.68-2622.15) | 2.3 (1.63-3.03) | -0.05 (-0.23-0.19) |
| 30-34 | 344864.51 (238402.91-447443.51) | 284.65 (196.78-369.32) | -0.04 (-0.21-0.2) |  | 5330.96 (3803.62-7048.89) | 4.4 (3.14-5.82) | -0.04 (-0.23-0.21) |
| 35-39 | 484908.51 (337020.43-634777.13) | 457.62 (318.05-599.05) | -0.09 (-0.26-0.14) |  | 8328.09 (5899.15-11040.34) | 7.86 (5.57-10.42) | -0.1 (-0.29-0.14) |
| 40-44 | 667893.01 (456843.95-886524.97) | 729.67 (499.1-968.52) | -0.11 (-0.3-0.14) |  | 12848.22 (8866.35-17229.78) | 14.04 (9.69-18.82) | -0.13 (-0.33-0.14) |
| 45-49 | 991067.64 (656180.83-1350530.7) | 898.34 (594.79-1224.18) | -0.17 (-0.36-0.08) |  | 20906.46 (13855.55-28961.51) | 18.95 (12.56-26.25) | -0.19 (-0.39-0.06) |
| 50-54 | 1631694.08 (1034041.71-2264126.21) | 1350.08 (855.58-1873.36) | -0.23 (-0.4--0.01) |  | 38720.75 (24265.41-53790.7) | 32.04 (20.08-44.51) | -0.26 (-0.44--0.03) |
| 55-59 | 1972818.86 (1184212.42-2733057.61) | 1794.41 (1077.12-2485.9) | -0.23 (-0.4-0) |  | 52773.89 (31437.96-74140.58) | 48 (28.59-67.44) | -0.27 (-0.45--0.02) |
| 60-64 | 1828703.04 (1029914.66-2607166.13) | 2504.9 (1410.74-3571.21) | -0.13 (-0.31-0.1) |  | 57204.63 (32803.75-81226.44) | 78.36 (44.93-111.26) | -0.17 (-0.34-0.09) |
| 65-69 | 2433916.97 (1279707.59-3672446.85) | 3173.15 (1668.38-4787.85) | -0.11 (-0.28-0.13) |  | 89886.23 (47826.45-134044.9) | 117.19 (62.35-174.76) | -0.14 (-0.32-0.11) |
| 70-74 | 2347045.95 (1131415.8-3734777.24) | 4403.76 (2122.87-7007.55) | -0.02 (-0.19-0.23) |  | 106697.38 (52122.36-168312.36) | 200.2 (97.8-315.8) | -0.05 (-0.23-0.21) |
| 75-79 | 1885150.76 (859808.86-3098664.76) | 5692.06 (2596.12-9356.17) | 0.06 (-0.11-0.3) |  | 108087.81 (50155.87-175755.55) | 326.36 (151.44-530.68) | 0.04 (-0.15-0.29) |
| 80-84 | 1709691.62 (784029.56-2850466.79) | 8638.36 (3961.37-14402.22) | 0.21 (0.03-0.45) |  | 128298.24 (58726.85-213157.3) | 648.24 (296.72-1077) | 0.2 (0.01-0.45) |
| 85-89 | 1270095.84 (584468.26-2137448.68) | 13333.29 (6135.67-22438.64) | 0.35 (0.16-0.59) |  | 122665.01 (56977.07-205914.39) | 1287.72 (598.14-2161.66) | 0.35 (0.15-0.6) |
| 90-94 | 548432.01 (260858.06-901372.07) | 18705.13 (8896.97-30742.7) | 0.31 (0.13-0.53) |  | 61598.37 (29360.62-101406.29) | 2100.91 (1001.39-3458.62) | 0.3 (0.12-0.53) |
| 95+ | 147040.35 (72304.78-241364.89) | 23007.46 (11313.56-37766.46) | 0.24 (0.05-0.47) |  | 17550.22 (8683.23-28736.75) | 2746.09 (1358.67-4496.45) | 0.25 (0.05-0.49) |

DALYs, disability-adjusted life years; TPC, total percentage change

Supplementary Table 5 The burden of DALYs and death of patients with MASLD in 2021 in China, with percentage changes from 1990 to 2021

| Age | DALYs (95% UI) | | |  | Mortality (95% UI) | | |
| --- | --- | --- | --- | --- | --- | --- | --- |
|  | Number | Rate | TPC (1990-2021) |  | Number | Rate | TPC (1990-2021) |
| ＜5 | - | - | - |  | - | - | - |
| 5-9 | - | - | - |  | - | - | - |
| 10-14 | - | - | - |  | - | - | - |
| 15-19 | 829.35 (565.56-1152.34) | 1.11 (0.76-1.54) | 0 (-0.18-0.2) |  | 10.88 (7.48-14.92) | 0.01 (0.01-0.02) | -0.01 (-0.19-0.2) |
| 20-24 | 1424.54 (999.51-2010.86) | 1.95 (1.37-2.75) | 0.04 (-0.2-0.29) |  | 20.41 (14.27-28.73) | 0.03 (0.02-0.04) | 0.03 (-0.21-0.3) |
| 25-29 | 2933.74 (1995.62-4259.56) | 3.39 (2.31-4.93) | -0.01 (-0.19-0.2) |  | 45.93 (31.13-66.54) | 0.05 (0.04-0.08) | -0.02 (-0.19-0.2) |
| 30-34 | 7752.65 (5382.46-10963.16) | 6.4 (4.44-9.05) | 0 (-0.18-0.21) |  | 132.41 (91.92-187.75) | 0.11 (0.08-0.15) | 0 (-0.18-0.21) |
| 35-39 | 11491.17 (7736.15-16780.3) | 10.84 (7.3-15.84) | 0 (-0.21-0.25) |  | 214.49 (144.72-314.05) | 0.2 (0.14-0.3) | -0.01 (-0.22-0.25) |
| 40-44 | 17581.45 (12035.93-24912.39) | 19.21 (13.15-27.22) | 0 (-0.24-0.29) |  | 363.58 (248.46-514.56) | 0.4 (0.27-0.56) | 0 (-0.25-0.29) |
| 45-49 | 32538.99 (22586.62-46563.2) | 29.49 (20.47-42.21) | -0.04 (-0.27-0.28) |  | 751.17 (521.82-1078.56) | 0.68 (0.47-0.98) | -0.04 (-0.28-0.28) |
| 50-54 | 50193.54 (33959.88-72585.18) | 41.53 (28.1-60.06) | -0.04 (-0.28-0.26) |  | 1301.81 (880.75-1886.27) | 1.08 (0.73-1.56) | -0.04 (-0.28-0.26) |
| 55-59 | 55662.56 (37406.46-80114.08) | 50.63 (34.02-72.87) | 0 (-0.23-0.31) |  | 1638.55 (1102.82-2351.98) | 1.49 (1-2.14) | 0 (-0.23-0.31) |
| 60-64 | 48668.23 (34541.9-67795.15) | 66.66 (47.31-92.86) | -0.05 (-0.25-0.21) |  | 1668.97 (1182.87-2319.32) | 2.29 (1.62-3.18) | -0.05 (-0.25-0.21) |
| 65-69 | 62828.81 (44696.93-84670.98) | 81.91 (58.27-110.39) | -0.07 (-0.26-0.16) |  | 2553.66 (1813.56-3437.77) | 3.33 (2.36-4.48) | -0.07 (-0.26-0.17) |
| 70-74 | 49347.77 (34705.86-65990.78) | 92.59 (65.12-123.82) | -0.02 (-0.21-0.2) |  | 2431.44 (1709.95-3241.4) | 4.56 (3.21-6.08) | -0.02 (-0.21-0.2) |
| 75-79 | 33344.41 (23471.45-45346.27) | 100.68 (70.87-136.92) | -0.04 (-0.22-0.17) |  | 2057.25 (1449.79-2804.92) | 6.21 (4.38-8.47) | -0.04 (-0.22-0.17) |
| 80-84 | 23167.65 (16483.49-31219.95) | 117.06 (83.28-157.74) | -0.05 (-0.21-0.12) |  | 1828.45 (1297.41-2471.05) | 9.24 (6.56-12.49) | -0.06 (-0.21-0.12) |
| 85-89 | 12116.01 (8687.06-16112.81) | 127.19 (91.2-169.15) | -0.03 (-0.18-0.14) |  | 1201.53 (863.48-1606.1) | 12.61 (9.06-16.86) | -0.03 (-0.18-0.14) |
| 90-94 | 3864.76 (2622.58-5580.51) | 131.81 (89.45-190.33) | -0.01 (-0.15-0.16) |  | 440.09 (296.79-637.63) | 15.01 (10.12-21.75) | -0.01 (-0.15-0.16) |
| 95+ | 725.62 (417.99-1165.93) | 113.54 (65.4-182.43) | -0.03 (-0.16-0.14) |  | 87.74 (50.64-141.6) | 13.73 (7.92-22.16) | -0.03 (-0.16-0.14) |

DALYs, disability-adjusted life years; TPC, total percentage change
